# A Persuasive and Social mHealth Application for Physical Activity: A Usability and Feasibility Study

**DOI:** 10.2196/mhealth.2902

**Published:** 2014-05-22

**Authors:** Soleh U Al Ayubi, Bambang Parmanto, Robert Branch, Dan Ding

**Affiliations:** ^1^Health and Rehabilitation Informatics LaboratoryDepartment of Health Information ManagementUniversity of PittsburghPittsburgh, PAUnited States; ^2^Department of InformaticsSchool of Electrical Engineering and InformaticsBandung Institute of TechnologyBandungIndonesia; ^3^Department of MedicineUniversity of PittsburghPittsburgh, PAUnited States; ^4^Department of Rehabilitation Science and TechnologyUniversity of PittsburghPittsburgh, PAUnited States

**Keywords:** mobile applications, mHealth, self-management, social support, persuasion, physical activity, usability, feasibility studies, pedometer

## Abstract

**Background:**

Advances in smartphones and the wide usage of social networking systems offer opportunities for the development of innovative interventions to promote physical activity. To that end, we developed a persuasive and social mHealth application designed to monitor and motivate users to walk more every day.

**Objective:**

The objectives of this project were to conduct a focused review on the fundamental characteristics of mHealth for physical activity promotion, to develop an mHealth application that meets such characteristics, and to conduct a feasibility study to deploy the application in everyday life.

**Methods:**

This project started as an analytical study to review the fundamental characteristics of the technologies used in physical activity monitoring and promotion. Then, it was followed by a technical development of the application. Next, a 4 week deployment was conducted where participants used the application as part of their daily life. A think-aloud method and in-depth semistructured interviews were conducted following the deployment. A qualitative description method was used to thematically analyze the interviews. Feasibility measures included, adherence to the program, user-system interactions, motivation to use, and experience with physical activity and online social interactions.

**Results:**

There were seven fundamental characteristics of physical activity monitoring and promotion that were identified, which were then used as a foundation to develop the application. There were fourteen participants that enrolled in the application evaluation. The age range was from 24 to 45; body mass index ranged from 18.5 to 42.98, with 4 of the subjects falling into the category “obese”. Half of them were experienced with smartphones, and all were familiar with a social network system. There were thirteen participants that completed the study; one was excluded. Overall, participants gave high scores to almost all of the usability factors examined, with averages of 4.52 out of a 5.00 maximum. Over 29 days, participants used the application for a total of 119,380 minutes (average=7.57 hours/day/participant; SD 1.56).

**Conclusions:**

Based on the fundamental characteristics, the application was successfully developed. The usability results suggest that the system is usable and user satisfaction was high. Deploying the application was shown to be feasible for the promotion of daily physical activity.

## Introduction

### Physical Activity and Smartphones

Despite the numerous proven benefits of physical activity (PA) [1-3] and widely publicized exercise guidelines [4,5], only 38% of adults in the United States engaged in regular leisure-time PA, and at least 25% were completely inactive [6]. Furthermore, most individuals who do begin exercise programs do not continue [7]. To promote PA, we need a system that meets the following two requirements: (1) complies with established health behavior change theories and strategies, and (2) is able to deliver effective and innovative interventions. At this moment, simple guidelines informing people of how to meet these requirements are not available. Fortunately, on the other hand, technological advances in smartphones offer innumerable opportunities for the development of such interventions as using them to monitor PA, and to encourage people to engage in more PA. As a result, a number of mobile applications (app) for PA have been developed. Unfortunately, most of these apps were not developed based on established health behavior change theories and evidence-based strategies, such as reinforcement and goal setting [8], which are keys to successful PA interventions [8,9].

### Persuasive Social Network for Physical Activity

We thus reviewed and analyzed a theoretical foundation for a PA monitoring and promotion system that complies with behavior change theories and strategies. Then we used the result from the previous step to develop a mHealth app called Persuasive Social Network for Physical Activity (PersonA). Technically, PersonA was designed to automatically receive raw data from PA sensors, calculate the data into meaningful information, store the information on a secure server, and show the information to the users as persuasive and real time feedback, or publish the information to a social networking system (SNS) for further social support purposes. PersonA was also designed to persuade users to have more PA by taking more steps every day. Increasing PA by taking more steps was chosen mainly for the fact that walking and running are the easiest, safest, cheapest, and most common PA for the general population, and at the same time, yield positive overall health outcomes [10-12]. It was also inspired by a public acceptance guideline of “10,000 steps/day” as a benchmark for an active lifestyle [13].

PersonA incorporates important features from previous projects or commercial products available on the market, including automatic data collection (as done by UbiFit, It’s LiFe!, Fitbit, BodyMedia, Actigraph, RunKeeper, Endomondo, and iSmoothRun) [14,15], an aesthetically appealing interface for data display and feedback (as done by UbiFit, Fish’n’Steps, Flowie, Young & Active, Fitbit, BodyMedia, RunKeeper, Endomondo, iSmoothRun, Actigraph) [14,16-18], social comparison and display of information trends (as done by UbiFit, Fish‘n’Steps, Ambient Display, Wellness Partner, Fitbit, BodyMedia, Endomondo, and Actigraph) [14,16,19,20], and ease of integration into everyday life (as done by all these projects, Fitbit, BodyMedia, RunKeeper, Endomondo, and Actigraph) [14-21]. Nevertheless, PersonA introduced new features in not only allowing a sharing of data, but also facilitating advanced social interactions among users, such as sending greetings and messages, giving comments, and setting up challenges similar to the NBC reality television show *The Biggest Loser*. The interactions can take place between two individual members, among several group members, or with any social network friends. PersonA uses the smartphone’s accelerometer to generate PA information (as done by RunKeeper, Endomondo, and iSmoothRun) to replace additional devices, such as traditional pedometers (necessary for Fish'n'Steps, Flowie, and Chick Clique) [16,17,21] or extension data monitoring devices (necessary for UbiFit, Fitbit, BodyMedia, Nike+, and Actigraph) [14]. Given these main features, PersonA was not designed as an invasive and very accurate tool to measure PA; rather it was designed as an easy to use tool, even though it may not provide very accurate information.

This paper reports the review and analysis of a theoretical foundation for a PA monitoring and promotion system, the development of a PA monitoring and promotion system called PersonA, and the results from a usability and feasibility evaluation of the system. Here, mainly viewed from the technological perspective, the evaluation serves two purposes: (1) to identify whether the system is usable and accepted by users; and (2) to reveal other issues in the deployment of this technology that contribute to an informed preparation for clinical trials. To our knowledge, this is the first study conducted to incorporate all of the purposes listed above.

## Methods

### Review of Fundamental Characteristics of Physical Activity Monitoring and Promotion System

Prior to the technical design of PersonA, an analysis to establish a theoretical foundation for the development of a PA promotion system was conducted. The purpose of the analysis was to identify the technical characteristics of a PA promotion system that: (1) complies with established health behavior change theories and strategies, and (2) is able to deliver effective and innovative interventions. Compliance with the behavior change theories and strategies has been recognized as a key component of successful PA interventions [8,9]. The characteristics were distilled from the research literature on 11 fundamental theories and models related to health behavior change, six design principles of behavior change systems, and 27 studies deploying health behavior change. The theories and models include, the Health Belief Model (HBM) [22,23], the theory of reasoned action (TRA) / theory of planned behavior (TPB) [24,25], the Elaboration Likelihood Model (ELM) [26,27], the social cognitive theory (SCT) [28,29], the social support and health link theory [30], the uses and gratifications theory (UGT) [31,32], the common bond and common identity (CBCI) theory [33], the Technology Acceptance Model (TAM) [34], the Unified Theory of Acceptance and Use of Technology (UTAUT) [35], and the Fogg Behavioral Model (FBM) [36]. The six design principles are, the functional triad and design principle [36], the persuasion theories and information technology design [37], the eight-step design process [38], the persuasive system design [39], the framework for health behavior change through social media [40], and the five strategies for supporting healthy behavior change [41]. The characteristics were also distilled from 27 studies on health-based promotion programs [17,30,42-66].

From this set, the characteristics of the interventions or technologies discussed were identified. In creating a list of characteristics, we focused on the characteristics that: (1) have an apparent relationship with the success of the health behavior intervention, (2) have the potential to be applied broadly across the health and wellness domain, especially PA promotion, and (3) have the potential to be leveraged in technologies currently available.

### PersonA Development

#### Sensing Technology, Smartphone, and Social Network Platforms

To accommodate the required characteristics, as a result of the analysis described above, three technology platforms were utilized: (1) sensing technology, (2) smartphones, and (3) social network technology. It has been recognized that self-monitoring using manual data input can be cumbersome; data input by the subject is largely unreliable due to biases associated with retrospective recall [66-69]. As a result, low adherence to manual self-monitoring commonly occurs [63,64,68]. Sensing technologies offer an alternative to the manual self-measuring tools by providing reliable, comfortable, and automatic data collection. Currently, sensing technologies are available with multi-functions, small sizes, and low prices, and are anticipated to be in more than 400 million devices by 2014 [70]. In PersonA, we use an internal-phone movement sensor, called an accelerometer, which can be used to generate PA information such as, number of steps, distance travelled, average speed, and energy expenditure.

The use of smartphones for PA monitoring and encouragement is appealing for a number of reasons. First, they have widespread use; their usage has reached a critical mass, with market penetration in the United States reaching 55% in early 2013 [71]. Second, the smartphone’s constant proximity to the user means that users can perform self-management and social interaction at any time or place. The addition of positive social support from social networks can amplify the smartphone’s persuasive power. Third, the ongoing improvements in mobile computing power and Internet connection allow for a more sophisticated assessment, calculation, analysis, and intervention, which can be remotely processed on the device itself or on a server. Together with more convenient interaction features (eg, bigger screen size, touch screen), these advanced functions may result in an increase of adherence and quality of health behavior programs.

PersonA uses Facebook as a platform for social support and networking. Facebook is the most widely used SNS in the world, with 1.11 billion monthly active users and over 655 million daily active users [72]. We utilized Facebook’s social interaction functions that are open to third party apps. The third party can access Facebook functions through an open application-programming interface (API), called Graph API. The API provides almost all of the functions necessary for the online interactions used in PA promotion. These functions include posting feeds, giving comments, authentication, security settings, and privacy/confidentiality settings.

#### Architectural Design

**Figure 1:**
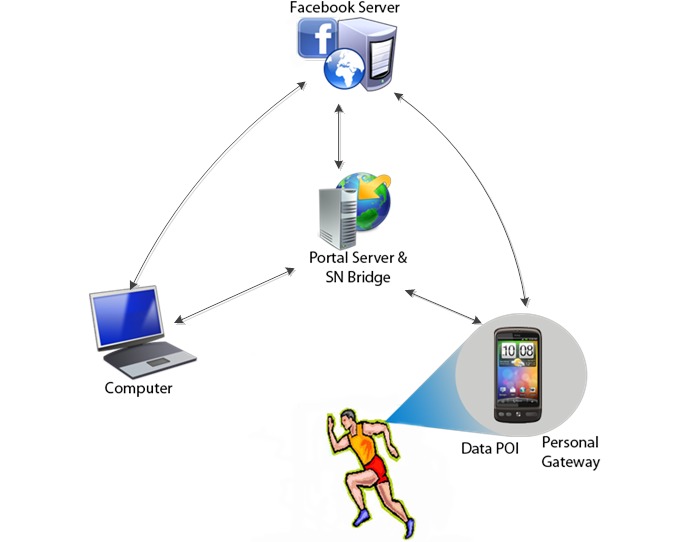
PersonA general architecture. POI: point of input; SNS: social networking system.

PersonA’s hardware architecture consists of the accelerometer sensor on an Android smartphone as a data point of input (POI), the Android smartphone as a personal gateway, portal server, SNS bridge, and Facebook infrastructure. The data POI detects and feeds PA data to PersonA. The personal gateway stores the sensory data temporarily, analyzes the sensory data, offers post analyzed and meaningful feedback on the smartphone app, and transmits the data to the remote portal server where the data will be stored. Because Hypertext Transfer Protocol is used in the data transmission from the personal gateway to the portal server, the gateway must have an Internet connection service such as General Packet Radio Service, 3rd generation (3G), 4th Generation (4G), or a wireless local area network. The portal server uses distributed database architecture to store the PA data, mapping it with user’s profile data. In addition to serving as a data repository, the portal server also acts as a Web server, hosting the PersonA engine system and Web services. The SNS bridge is a system connecting the portal server or personal gateway with the SNS (Facebook) server. The Android smartphone was chosen as a primary personal gateway because the Android Operating System (OS) is a free and open source, allowing apps to be easily developed on top of it, and is a predominant OS on smartphone devices [71]. PersonA was designed to work on any phone with an Android OS version 2.3 or higher. In this study, the Android smartphones used were the Samsung Droid Charge, Nexus S, and Nexus S 2. A majority of the phones used the Verizon Wireless service 4G. The 2010 Microsoft SQL Server Enterprise Edition was used as the database server and the Apache Tomcat 6.0 was used as the Web server. For data transmission among the components, a RESTful Web service with JavaScript Object Notation (JSON) data format was used. Figure 1 shows this architecture.

In addition to the mobile app, a Web app was developed with the exact same features, with the exception of data collection. The difference between the two apps lies in how often and extensive PA information is provided. The mobile app provides more immediate feedback than the Web app, while the Web app provides more extensive summaries and views of PA participation for individual users and group aggregates. These differences are mainly caused by the nature of the technologies (smartphone and computer) by which PersonA is accessed. The mobile phones are carried on the person, always turned on, personal, and portable; but are limited on computation power and screen size. On the other hand, computers have better computation power and larger screen sizes.

### Usability and Feasibility Evaluation

#### The Five Usability Factors

Usability testing is a technique utilized in user-centered interaction design to evaluate a product by testing it on users [73]. The testing is traditionally associated with these five usability factors: (1) learnability, the system should be easy to learn so that the user can rapidly start getting work done; (2) efficiency, it should be efficient to use so that the users, having learned the system, are able to perform their tasks productively; (3) memorability, it should be easy to remember so that the casual user is able to return to the system, after some period of not having used it, without having to learn everything all over again; (4) error recovery, it should have a low error rate so that users make few errors while using the system, and these errors are easy to recover from. Further, catastrophic errors must not occur. And (5) satisfaction, the system should be pleasant to use so that users are subjectively satisfied when using it [73].

To evaluate the five factors, the formative usability assessment generally utilizes the following three protocols: (1) think-aloud assessment; (2) post study questionnaire; and (3) in-depth semistructured interview. First, the think-aloud assessment (think-aloud protocols, or talk-aloud protocol) is a method used to gather data in usability testing where, while performing a test task, users are asked to talk about what they are thinking, what they are trying to do, voice questions that arise as they work, and ask about things they read. This protocol was first introduced in the usability field by Lewis [74], and then was explained in more detail in another work [75]. Second, the post study questionnaire was designed to evaluate the five usability factors quantitatively. A few researchers proposed a, “ready to use tool” of post study questionnaires that all refer to the Nielsen work [73].

In this PersonA study, during the development phase, we conducted two in-lab usability tests to identify problems on the app, and to increase the performance. Participants of the test were four researchers and two potential users. The results from this initial usability test were used to iteratively refine PersonA. After PersonA had been successfully developed and tested without any critical errors or problems with usability, an everyday life usability and feasibility evaluation was conducted, from which the results are presented in this paper. The purpose of the evaluation was to assess whether PersonA is usable and easy to utilize by users, as well as to assess whether PersonA can be deployed in real, everyday life settings. The University of Pittsburgh Institutional Review Board (IRB PRO12020634) approved the evaluation.

#### Participant and Recruitments

To evaluate the usability and feasibility of PersonA, potential users were recruited through paper pamphlets and a Web-based advertisement (Facebook page). Potential users were included if they were: (1) 18-65 years of age, (2) able to operate a computer and smartphone, and (3) able to walk or run without difficulty. The exclusion criteria were: (1) inability to tolerate sitting for 2 hours or more, (2) history of cardiovascular disease, and (3) history of breathing problems and/or respiratory disease with associated breathing problems. Participants were compensated US $50.00 for taking part in the study. The participant sample size of evaluation was determined using the Problem Discovery Rate Model [76-78] that has been widely used to serve formative evaluations. The model estimates that 85% of usability problems will be revealed using five participants, and almost 100% of problems will be revealed using 14 participants [79-81].

#### Study Design and Procedure

Participants started taking part at the end of the development phase of PersonA. Participants were invited to two 2-hour visits at the University of Pittsburgh. At the first visit, the purpose and overall procedures of the study were explained to the participants. After signing a consent form, participants were asked to complete two questionnaires eliciting demographic information and experience with the Internet, smartphones, and social networking sites. Then, a brief orientation and demonstration on how to use PersonA was provided. After the orientation, participants were sent home and asked to use PersonA daily for four weeks. A smartphone with an unlimited data plan was provided to each participant. During the four week study period, the built-in tracking function in PersonA was active to monitor all activities done within the app, including how much time participants spent using PersonA, how often they accessed PersonA, and which features of PersonA were most used. In order to explore whether online social interaction may associate with PA performance, we decided to have a pilot baseline intervention design. Therefore, to build a baseline for personal PA, the participants had no social interaction (social menu) in the first week; then the social menu was introduced in the beginning of the second week and was available until the end of the study. At the end of the fourth week, the participants were asked to come back to perform a number of tasks using a think-aloud method, then asked to complete a customized usability questionnaire. At the end of this process, participants were then asked to take part in an in-depth semi-structured interview. The interview served two purposes: (1) to clarify participants’ answers on the usability questionnaire, if necessary, and (2) to answer several questions related to the feasibility evaluation, especially those related to participants’ experiences during the study period. This interview was recorded as an audio and video format for transcription and further data analysis.

#### Outcome Measures

##### Usability

Usability data was distilled from the answers provided on the questionnaire and the interview. The questionnaire was focused on investigating usability factors adapted from the International Business Machine Post Study System Usability Questionnaire (PSSUQ) [82], Nielsen’s attribute of usability [73], and TAM [34]. These usability factors are learnability, efficiency, memorability, error recovery, and subjective satisfaction. The questionnaire also investigated one additional factor related to the technologies used in this study, especially smartphones. That factor is navigation, which is very important in smartphone apps, but was not included in the original PSSUQ, Nielsen’s attribute, or TAM.

##### Feasibility

Information on feasibility was obtained through the interview and the embedded function that tracked users’ activities with PersonA. No existing standardized or validated measurement tools or methods were used to obtain this information. There are several aspects of feasibility that were evaluated in this study, including participants’ adherence to the program, system usage, user-system interactions, participants’ preferences with regards to the systems, participants’ motivation to use PersonA, and participants’ experience with PA and online social interactions.

##### Persuasiveness

A variety of data was intentionally collected in order to explore the persuasiveness of PersonA. The tools used include, questionnaire, interview, user-system interaction, and PA data. To avoid confusion or a misunderstanding, the concept of persuasion in this manuscript always refers to the persuasive concept in the Computer Science field, not lifestyle behavior change, unless stated otherwise.

##### Pilot Physical Activity Data

There were four sets of objective PA data that were collected in this study: (1) number of steps, (2) energy expenditure, (3) distance traveled, and (4) average speed. The number of steps was obtained using the smartphone’s accelerometer sensors. These sensors were tested in a laboratory environment where two researchers put the smartphone in their front pants pocket, walking (and mentally counting) 400 steps in a flat area; this procedure was repeated 7 times. The sensors recorded an average of 392 (SD 13) steps. These results may be different in a free-living condition. Energy expenditure data was estimated based on a calculation of number of steps and body weight. Distance traveled was calculated based on the multiplication of the number of steps and step length. Average velocity was calculated based on the number of steps, step length, and the duration of system while in an active status of collecting data. Since the reliability and validity of the four PA datasets have not been evaluated, the data were neither highlighted nor included in the discussion or in the conclusion of this paper.

## Results

### PersonA Characteristics Model

#### Physical Activity Monitoring Systems and Health Behavior Change Theories

**Figure 2:**
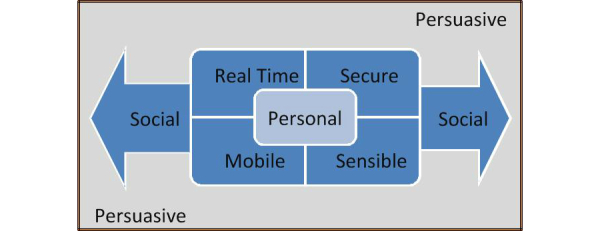
PersonA Characteristics Model.

As a result from the analytical study described above, we identified seven technical characteristics that a PA monitoring and promotion system must have to comply with the established health behavior change theories and strategies, as well as to be able to deliver effective and innovative interventions (Figure 2 shows these characteristics).

#### The Seven Characteristics

##### Personal

The system should be attached, or at least connected, to users whenever and wherever they are. It should also provide users ownership of their physical phenomena data, allowing them to decide with whom, and for what reason, their data will be shared. Moreover, the system should be able to deliver a personalized or tailored intervention, instead of a general or fit-for-all intervention.

##### Sensible

The system should give users the ability to collect their physical phenomena data easily (automatically or with minimum effort), and then store the data to an appropriate designated location with unobtrusive communication channels.

##### Real Time

The system should provide the necessary information that users need within milliseconds so that it is virtually available at the time it is needed.

##### Secure

The system should protect the confidentiality and privacy of users’ health and personal data. The protection should be applied at the start of the user/system data collection, storing processes, retrieving processes, and other processes, such as sharing with others.

##### Mobile

The system should be able to move easily and freely in tandem with the users.

##### Social

The system should support or provide users with the ability to compare their performance with that of others, to have companionship, and to have social interaction as part of their health behavior activities.

##### Persuasive

The system should be able to induce action, or foster belief, through reasoning, inspiration, or encouragement.

### The Seven Characteristics and Health Behavior Change Theories

The relationship between the seven characteristics and the established health behavior change theories, from which the characteristics are distilled, is illustrated in Table 1.

**Table 1:** PersonA characteristics and the established health behavior change theories which include, the HBM, the TRA / TPB, the ELM, the SCT, the social support and health link theory, the UGT, the CBCI theory, the TAM, the UTAUT, and the FBM.

PersonA characteristics	Distilled from
Personal	The theoretical construct of the behavioral intention of the TRA; the perceived behavioral control of the TPB; and the self-efficacy of the HBM and SCT
Sensible	The theoretical construct of self-efficacy in the HBM and SCT; and the perceived behavioral control in the TPB
Real time	The principles of self-efficacy and cue to action of the HBM; and the theoretical construct in the UGT, which tells that one important gratification for people to use technology is to get information
Secure	The principles of the supportive and environmental factors of the SCT; the principle of convenience of the UGT; and the perceived usefulness (acceptance) of the TAM and UTAUT
Mobile	The principles of perceived benefit, self-efficacy, and cue to action of the HBM; the perceived usefulness and perceived ease of use of the TAM; and the performance expectancy of the UTAUT
Social	The principles of the SCT, social support, the UGT, the common bond and common identity theory, and the social support and health link theory (which include the supportive environmental factor, influence of belief and cognition, social categorization, cooperative interdependence, intergroup comparison, social interaction, exchange of personal information, personal attraction through similarity, sense of belonging, social enhancement, and maintenance of interpersonal connectivity)
Persuasive	The principles of cues to action of the HBM; the self-efficacy of the HBM and SCT; the perceived behavioral control of the TPB; the central and peripheral routes of persuasion of the ELM, the entertainment and convenience of the UGT, the perceived ease of use of the TAM, the experience of the UTAUT, and the motivation and trigger of the FBM

### PersonA Application

#### PersonA Capabilities

##### Capability Descriptions

**Figure 3:**
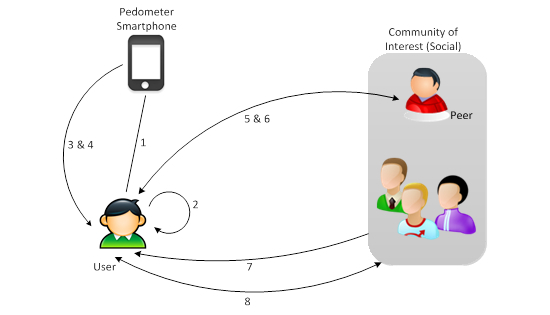
PersonA functional requirements. 1: self-measurement; 2: goal setting; 3: self-monitoring; 4: self-comparison; 5: peer support; 6: peer comparison - competition; 7: social support; 8: social comparison - competition.

It has been widely recognized that self-management and social support have a positive impact on PA participation [9,14-20,30,37,39,40,46,83]. Therefore, PersonA was also designed to accommodate users to perform self-management and social support practices [84]. Figure 3 illustrates PersonA’s capabilities that were designed to meet the self-management and social support requirements; while detailed descriptions for each capability follow.

##### Self-Management Functional Requirement

###### The Four Requirements

PersonA includes the four most important self-management requirements: (1) self-measurement, (2) goal setting, (3) self-monitoring, and (4) self-comparison.

###### Self-Measurement

Self-measurement allows expected PA data to be captured automatically using sensor devices and then transferred to a smartphone. Once the data is stored on the smartphone, it can be displayed as immediate and persuasive feedback. Alternatively, the data can be sent to the health portal server for further analysis or for display on the portal side. The automatic data collection can potentially increase users’ adherence to the PA program. It allows patients to measure their physical phenomena and to obtain reliable data with less dependency on health practitioners. Moreover, it reduces the users’ effort and is also more comfortable for them than manual data collection.

###### Goal Setting

Goal setting allows users to define a target that they want to reach. Using this capability, users can more easily set a realistic PA goal for a specific time. Before doing so, however, users can compare the new target with one that is already set, and a new default target is set automatically by PersonA. Comparing the three may encourage the user to set and reach a better goal every day.

###### Self-Monitoring

**Figure 4:**
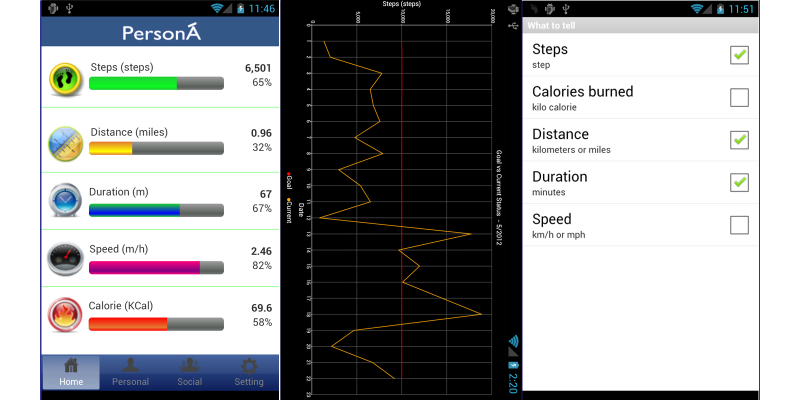
PersonA self-management features.

Self-monitoring helps users to monitor and compare a predefined goal against their current status. It also helps users to positively self-enforce a commitment to that predefined goal. The ideal scenario is that automatic and real time data collection is available along with immediate feedback so that users know how far they are from their target. The self-monitoring chart (Figure 4 shows the self-monitoring features-left) shows how users can easily check the actual value for each activity item while they are performing a physical task. They can also monitor the progress they make by looking at the progress bar for each item and its percentage count, all of which is displayed on the same screen. The progress bar is used in order to convey the user’s progress for PA tasks. For example, Figure 4 (left side of Figure) shows clearly that the user has reached 6501 steps, which is 65.01% (6501 actual steps/10,000 target steps) of the target.

###### Self-Comparison

Self-comparison allows users to monitor and compare their activity data over time. It provides a longitudinal chart that shows a comparison between a user’s target and the actual achievement; it also occasionally shows long-term trends or even dips and spikes (Figure 4-middle).

###### Encouraging Performance

Being able to monitor all of these activities may encourage users to perform better while engaged in PA. In relation to the implementation of the persuasive concept in the FBM, self-monitoring is part of an intrinsic strategy to persuade people to change behavior [36]. Using this strategy, PersonA was then designed to motivate users by triggering intrinsic personal drive, such as by setting goals, creating awareness, or by conditioning through positive reinforcement that may lead to increased PA.

In addition to visual feedback, mobile PersonA also provides aural feedback. This aural feedback is implemented because real time feedback is sometimes needed when users are performing PA, and it is difficult to view feedback on smartphones while moving. Users can set up which information they want to hear and at a specified frequency (Figure 4-right).

##### Social Support Functional Requirement

**Figure 5:**
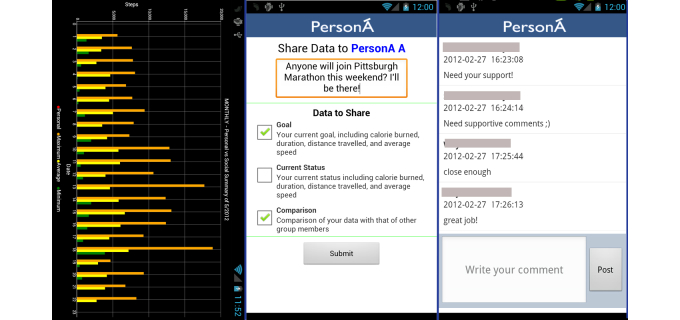
PersonA social support features.

Social-support requirements are designed to help users engage with peers or social networks that can positively affect their PA performance. PersonA provides four functional features to facilitate the peer and social interactions. First, the peer-comparison feature allows an individual to compare his/her performance with that of one person in the app. This allows a more personal comparison, especially with a peer who is personally known, such as a close friend or spouse. Second, the group-comparison feature, which allows an individual to compare his/her current PA performance and target with the group average, the larger community average, or the normal standard set by health practitioners. Figure 5 illustrates a chart that compares the summary of a user’s caloric expenditure with that of the social network (left). The chart also provides the comparison longitudinally. Third, the peer-support feature that allows individuals to support each other with one peer in a closed interaction where the individual and her/his peer only can see and communicate using this channel. Fourth, the group-support feature that allows users to support each other in open interaction where every member of the group can see and interact. While using the above four features, the following positive support activities can be done by users: (1) giving rewards or greetings for reaching a goal, (2) sharing experiences or activities, and (3) “liking” others’ status or data. The user can choose to share data with a friend, a member group, or even all friends on Facebook. As an illustration, Figure 5 (middle) shows that users can share their selected data with members of a Facebook group. As with other standard posts on a Facebook wall, these posts can be liked or commented upon by friends of users. PersonA also provides users with a message archive where the users can access all related communication that they made using PersonA, and perform further social interaction (Figure 5-right).

As inspired by other studies finding that social interaction had a positive effect in increasing PA performance [14,16,17,19,21], or at least it did reduce participant attrition even though it did not increase average PA performance [83], PersonA was designed to have the above social feature, which may boost users’ performance and increase the likelihood of their adherence to the program. In relation to the persuasive concept, the social-comparison and peer-support are part of an extrinsic strategy to persuade people to change behavior [36]. Using this strategy, PersonA was designed to motivate users to build on the social psychology where other people are the source of the motivation, for example, through competition, cooperation, or comparison, which may finally lead to increased PA.

#### Persuasiveness

To increase the persuasiveness of PersonA in encouraging people to perform more PA, we addressed the following methods. First, since the integration increases the likelihood of a system to be adopted [36,55], PersonA was integrated with an app that has demonstrated to have psychological and social value to the users. PersonA was bundled with the most widely used SNS, Facebook. Second, the PersonA interface is designed to be as interactive as possible, where interactive experiences that are easily accessible and convenient have greater persuasive effects [36,55,85]. Third, PersonA is designed to have simple tasks, which may increase a user’s adherence to a health promotion program [36,55]. For example, automatic input in PersonA is simpler than paper-pencil or manual typing input. Fourth, in order to achieve an optimal result, PersonA will trigger users’ attention when they are most open to persuasion [36,55], by designing a system that gives immediate feedback, reminders, and greetings at opportune moments according to users’ preferences, health professional recommendations, or specific contextual information.

To illustrate more detail about the persuasive methods implemented in PersonA, the Persuasive System Design (PSD) framework [39] was used to classify the PersonA features by its persuasive functions. The PSD framework classifies the persuasive functions of a technology as primary task support, dialogue support, social support, and credibility support [25]. By using the PSD framework, we can systematically look at how all PSD elements are implemented in PersonA. The relation between the PersonA features and PSD elements is illustrated more detail in the Table 2.

**Table 2:** PSD framework elements and PersonA features.

Principle and definition according to PSD framework [39]					PersonA features
**Primary task support**					
			Reduction	A system reduces complex behavior into simple tasks that help users perform the target behavior, and it may increase the benefit/cost ratio of a behavior	Once a user turns on the PersonA app, PA data is collected automatically
			Tunneling	Using the system to guide users through a process or experience provides opportunities to persuade along the way	Goal setting pop-up message appeared every morning for daily goal, every Sunday morning for weekly goal, and the 1st morning of the month for monthly goal
			Tailoring	Information provided by the system will be more persuasive if it is tailored to the potential needs, interests, personality, usage context, or other factors relevant to a user group	The default value of goal is determined based on the user goal and performance from the previous day, week, and month
			Personalization	A system that offers personalized content or services has a greater capability for persuasion	The default value of goal is determined based on the user goal and performance from the previous day, week, and month
			Self-monitoring	A system that keeps track of one’s own performance or status supports the user in achieving goals	A user is able to monitor and compare a predefined goal against their current status, which eventually may positively self-enforce a commitment to the goal
			Simulation	A system that provides simulations can persuade by enabling users to observe immediately the link between cause and effect	-
			Rehearsal	A system that provides means with which to rehearse a behavior can enable people to change their attitudes or behavior in the real world	-
**Dialogue support**					
			Praise	By offering praise, a system can make users more open to persuasion	-
			Rewards	A system that rewards target behaviors may have great persuasive powers	Reward message will appear when users achieve certain percent of their target or achieve certain level (eg, top 10%) of the groups
			Reminders	If a system reminds users of their target behavior, the users will more likely achieve their goals	Users can setup a reminder to do PA
			Suggestion	A system offering fitting suggestions will have greater persuasive powers	The default value of goal is determined based on the user goal and performance from the previous day, week, and month
			Similarity	People are more readily persuaded through a system that reminds them of themselves in some meaningful way	Users can setup a reminder to do PA at their convenience
			Liking	A system that is visually attractive for its users is likely to be more persuasive	Users are able to see the information of their performance in real time. The information might be displayed in a stratified interface such as a garden or aquarium or a simple progress bar
			Social role	If a system adopts a social role, users will more likely use it for persuasive purposes	-
**Social support**					
			Social learning	A person will be more motivated to perform a target behavior if (s) he can use a system to observe others performing the behavior	Users are able to compare his/her performance with that of one person using the app. This is a generally more closed and intimate comparison, especially with a peer who is personally known, such as a close friend or spouse. In addition, users are able to compare his/her performance with the group average, the larger community average, or the normal standard set by health practitioners
			Social comparison	System users will have a greater motivation to perform the target behavior if they can compare their performance with the performance of others	
			Normative influence	A system can leverage normative influence or peer pressure to increase the likelihood that a person will adopt a target behavior	-
			Social facilitation	A system user is more likely to perform target behavior if they discern via the system that others are performing the behavior along with them	One user is able to support another user with one peer in a closed interaction where the individual and her/his peer only can see and communicate using this channel. Moreover, users are also able to support each other in open interaction where every member of the group can see and interact. These closed and open interactions might drive a competition
			Cooperation	A system can motivate users to adopt a target attitude or behavior by leveraging human beings’ natural drive to cooperate	
			Recognition	By offering public recognition for an individual or group, a system can increase the likelihood that a person/group will adopt a target behavior	
			Competition	A system can motivate users to adopt a target attitude or behavior by leveraging human beings’ natural drive to compete	

#### Security and Confidentiality

Security and confidentiality in a health app is of paramount importance; thus, we implemented the following methods to ensure that communication is secure and confidential. First, the authentication process requires a combination of the device’s phone number, the International Mobile Equipment Identity number, the email address, and a Facebook account. Only devices with a proper and registered combination will be able to push data to PersonA and access information from PersonA. However, the Web version of PersonA uses only a combination of an email address and a Facebook account to authenticate users who want to access the information. Second, the communication framework of PersonA handles the encryption and authentication process. Third, all infrastructures were hosted at the tertiary care center behind a firewall in a network secure environment. Fourth, by default, personal health data will be privately protected and just for personal access; but summary data, such as maximum/minimum/average data, will be available for all members of the PA promotion group.

**Figure 6:**
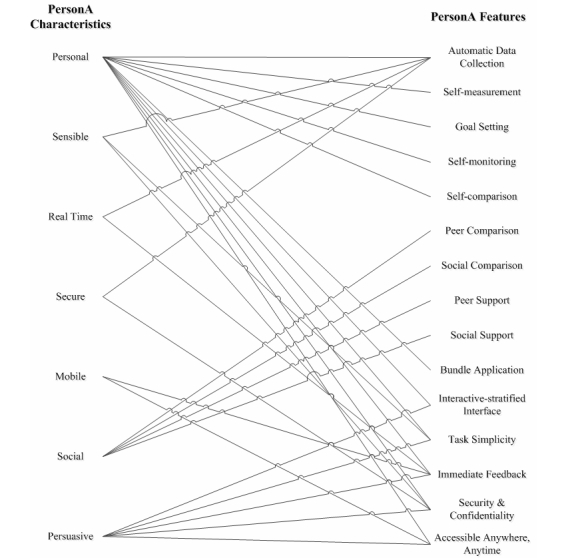
Relationships between PersonA Characteristics Model and PersonA features.

Finally, all above features were successfully developed based on the PersonA Characteristics Model. Figure 6 illustrates the relationship between the model and the features.

### Qualitative Measure Results

#### The Usability and Feasibility Evaluations

Qualitative measures from the usability and feasibility evaluation result are presented first, followed by results from the quantitative data. Due to the nature of this study, all quantitative data was analyzed mainly through a descriptive means rather than by hypothesis testing. There were fourteen potential users that were recruited, with thirteen participants completing the study. There were two participants that were overweight and four that were obese according to the body mass index (BMI) formula with self-reported body height and weight parameters. Another type of subjective information collected was “what type and in what frequency of intended PA that participants regularly do” which is then referred as PA habit in Table 3. The detailed self-reported demographic data is provided in Table 3.

**Table 3:** General demographic, PA habit, smartphone experience, and SNS experience.

Participant	Gender	Age	BMI	PA habit	Smartphone experience	SNS experience
P01	F	33	23.3	Jogging once a week and exercise intended walking 2-3 times a week	No experience	Several times a day, for more than 3 years
P02	F	40	30.1	None	No experience	Several times a day, for 2-3 years
P03	F	32	22.5	Occasionally	No experience	Several times a day, for more than 3 years
P04	F	35	42.9	Occasionally	Less than 1 year	Several times a day, for more than 3 years
P05	F	25	30.1	Twice a week (jogging, cycling, rowing, and strength training)	No experience with smartphone	Several times a day, for more than 3 years
P06	F	24	34.6	None	No experience	Once a day, for more than 3 years
P07	F	45	22.3	3-4 times a week (treadmill, elliptical, zumba/latin heat, weight lifting)	1-2 years	Several times a day, for 2-3 years
P08	F	30	21.3	2-3 times a week tennis and jogging; 5 times a week stretches	1-2 years	Regularly log on, for more than 3 years
P09	F	31	18.6	3 times a week jogging	No experience	Regularly log on, for more than 3 years
P10	F	30	26.8	Walking once a week, jogging once in two weeks	1-2 years	Several times a day, for 2-3 years
P11	M	34	24.2	Once a week running and swimming	2-3 years	Regularly log on, for more than 3 years
P12	M	29	26.6	Twice a week running	More than 3 years	Regularly log on, for more than 3 years
P13	M	30	24.1	2 times a week running and tennis, and 3 times a week swimming	6 months-1 year	Several times a day, for 2-3 years

#### Overall Usability Score

Overall, participants gave high scores to almost all usability factors, with an average of 4.52 of a 5.00 maximum. A breakdown of the numbers for each factor asked about is presented in Table 4.

#### Accuracy

Participants gave various scores for “accuracy” and scores of overall usefulness; and provided various comments on willingness to use the system when it was available. When asked to estimate the percentage of total steps captured daily by PersonA (sometimes they did not have the phone with them), answers varied widely, as can be seen in Table 5.

#### Usefulness

Most participants thought that the mobile app was very useful or extremely useful. One participant stated it was moderately useful, as can be seen in Table 6.

#### Willingness to Use

When participants were asked whether they would use the system when it becomes available, most of them expressed a willingness to use it, as can been seen in Table 7.

**Table 4:** Quantitative results for overall usability.

Usability factors (1=totally disagree, 5=totally agree)	Average (SD)
It was easy to learn how to use this system.	4.72 (0.33)
It was easy and simple to use this system.	4.69 (0.33)
It was easy to obtain what I need.	4.67 (0.32)
The interface of this system is pleasant.	4.35 (0.20)
I like the interface of this system.	4.41 (0.20)
The organization of information was clear.	4.38 (0.30)
It was easy to navigate to find what I need.	4.38 (0.22)
Whenever I made a mistake using the system, I could recover easily and quickly.	4.29 (0.36)
The system gave error messages that clearly told me how to fix problems.	4.29 (0.40)
This system has all the functions and capabilities I expected it to have.	4.67 (0.33)
Overall, I am satisfied with the quality of the service/information being provided via this system.	4.87 (0.28)
Average	4.52

**Table 5:** Percent of steps captured using PersonA.

Estimated % of steps captured	Number of participants
> 80.00	3
> 60.00 and ≤80.00	7
> 40.00 and ≤ 60.00	2
> 20.00 and ≤ 40.00	1
< 20.00	0

**Table 6:** Reported usefulness level.

Level of usefulness	Number of participants
Extremely useful	6
Very useful	6
Moderately useful	1
Slightly useful	0
Not useful	0

**Table 7:** Reported willingness to use PersonA when available.

Level of usefulness	Number of participants
Definitely use	7
Probably use	5
Not sure	1
Probably not use	0
Definitely not use	0

#### Estimating Steps Taken

Although perceptions varied, participants thought that PersonA was useful because it provided a good estimation of the number of steps taken. Typical comments included,

The apps may not give a very accurate step number, but it perfectly cues the estimation range...

P01

...I am more interested in relative numbers than absolute numbers. I wanted to know if I walked more today than what I did yesterday...

P05

It’s still useful even with this current level accuracy...

P07

When asked in which ways PersonA was useful, participants recorded a variety of answers, shown below in Table 8.

**Table 8:** Reported usefulness factors.

Usefulness factors	Number of participants
Making new friends	0
Self-monitoring PA levels by comparing current and target level	12
Knowing the activity levels of others or aggregate of the group	6
Comparing your activity with others	6
Finding people to exercise together	3
Sharing experience with others	3
Supporting each other	5
Finding useful information about PA	1

#### Motivation to Use

There were three themes of motivation to use PersonA that emerged from the qualitative sampling of participants’ comments. There was one motivation that wanted to know more about the number of steps taken throughout a day, inside and outside the gym. Especially those having poor or fair daily PA levels expressed this motivation. A typical response was,

...I want to know how many total steps that I have throughout a day.

P02

Another motivation participants expressed was to balance calorie intake-outtake. Especially those having good or very good daily PA levels expressed this motivation. Typical comments include,

I was interested knowing more about my energy expenditure. I was curious to see how many more calories that I burned outside the gym...Simply because I care whether my total calorie intake-outtake is balanced or not.

P05

The last motivation that emerged was being curious about how social interaction influences PA habits,

I was curious to know whether the social aspect of PersonA would change my exercise habit or not. At the end of the day, indeed, it changed my habit...

P03

#### Suggestions for Improvement

There were three themes that arose when participants were asked for suggestions to improve PersonA. The first theme is a suggestion to include other types of PA,

That would be nice if it includes other kind of activities, not just running and walking; like cycling and rowing.

P05

The second theme was to resolve the battery problem,

The battery is a problem because it lasts for only 5 hours.

P06

The last theme was to have smaller devices,

If possible, I want the apps [PersonA] running on smaller phone [not a smartphone]. It’s a lot easier to carry or put it in the pocket.

P01

#### Mobile Applications Over Web Applications

PersonA has two versions: (1) mobile app, and (2) Web app [84]. A comparison between these two versions was conducted to evaluate participants’ preferences between the Web and mobile versions. All of the participants chose the mobile version, typical comments included,

I like the smartphone one because I’m more likely to see the data on smartphone, for example while waiting for the bus or even on the bus. When I access a computer, I have so many other things to do, like working, checking email, etc. I would forget to access the apps [on the Web].

P05

I like the smartphone version. It’s easy to check the data. We have everything on our fingers; I don’t really need the Web version. What I have in my smartphone version is more than enough.

P03

I prefer the mobile simply because I bring the phone whenever and wherever I go...

P01

#### Online Social Interaction

Participants’ responses with regards to online social interaction revealed that PersonA may leverage this interaction to improve PA in a variety of ways, and on many levels, as presented in the following few comments.

There was one participant that expressed that social interaction may not work for her or some other people,

I have my own personal plan and personal schedule, so I never compared and never wanted to be encouraged to do walking. I know that I need to do physical activity; I know 10,000 steps per day [guidelines] is good for me and definitely I will do it when I have time...

P07

A tool equipped on PersonA for comparing user data to a target or to others’ performances may increase motivation to do more PA in some participants. The participants also said that online social interaction indirectly encouraged them to do more PA, at least in the sense that they see there is company while doing so,

A function to compare my physical activity with that of my friends is really nice. It maps myself in the group as well as informs that there are other people doing the same thing, so that I feel I’m not alone in doing it.

P02

Even though the social interaction feature is mainly intended to provide social support, some participants utilized the feature as a means of self-motivation,

...I only post my physical activity data on my own wall, because it’s more like to tell myself that I have to work out today. It’s used to remind myself.

P05

Other participants recognized the real effect of online social interaction in encouraging and improving levels of PA,

The social interaction changed my walking behavior. I still remember about two months ago, when I wanted to meet a friend in Pitts [about 3 miles from her home], I usually took a bus that sometime takes about 30 minutes, including waiting time. After using PersonA, I don’t do it anymore because of knowing that some people on the group have thousands of steps more than me. So, right now, I just walk to have more number on my apps... and, you know what makes me feel better, it turned out I only need about 20 minutes to get there! It saves time and makes me feel healthy.

P03

#### User-System Interaction

Over the 29 days of the study, participants used PersonA for a total of 119,380 minutes (average=454 minutes=7.57 hours/day/participant with SD 1.59 hours), and they accessed various system features an average of 28 times/day/participant. In addition to evaluating the overall usage, another purpose of collecting user-system interaction data was finding which PersonA features were used the most. As Table 9 shows, it seems that accessing personal data was favored over social interaction.

**Table 9:** User-system interaction per week.

Action	Week 1	Week 2	Week 3	Week 4	Total
Accessing home^a^	1046	1194	1025	1563	4828
Accessing personal data^b^	548	889	683	526	2646
Social interaction^c^	0	356	498	261	1115
Accessing goal or changing goal^d^	106	434	428	256	1224
App setting^e^	215	135	113	106	569

^a^Accessing home represents how many times the participants accessed the home page of PersonA. This number also represents the frequency of users’ access to the PersonA since the home page is the first page loaded when accessing the app.

^b^Accessing personal data represents how many times the participants viewed personal PA information. This information is a comparison between actual performance and target for the selected day, one day before, the current week, the current month, and total period since the participant began using the app.

^c^Social interaction represents how many times the participant utilized social interactions, including social comparison and social support. Social comparison includes sharing data with a friend, a group member, or even all friends on Facebook. It also includes equating their PA performance and target with those of others in the group, the group average, the larger community average, or the normal standard set by health practitioners. Social support activities include giving rewards or greetings for reaching a goal, sharing experiences or activities, and “liking” others’ status or data.

^d^Accessing goal and changing goal or target represents how many times users set up and reviewed their daily, weekly, or monthly goals.

^e^App setting represents how many times users set up the app. This includes setting email, setting body weight, setting or changing sensitivity of the accelerometer sensor, setting or changing PA type, running or walking, and setting or changing theme, only in Web version.

#### Physical Activity and Usage Data- Comparison Between Baseline and Social Intervention

**Figure 7:**
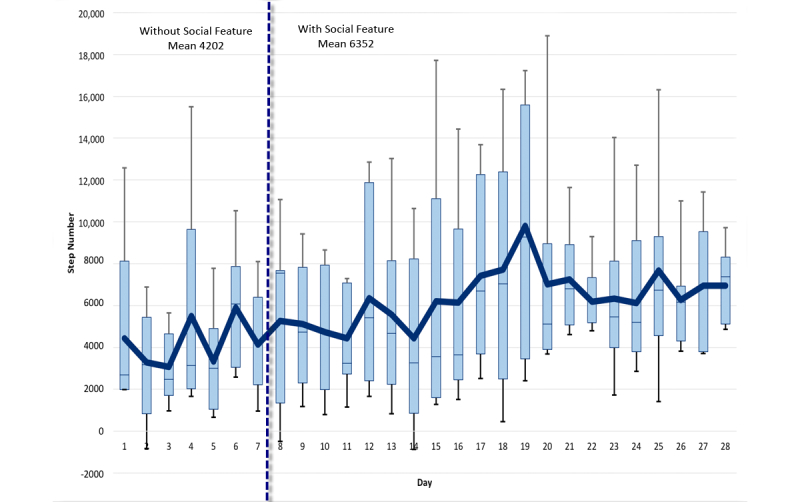
Steps comparison between baseline and social intervention.

**Figure 8:**
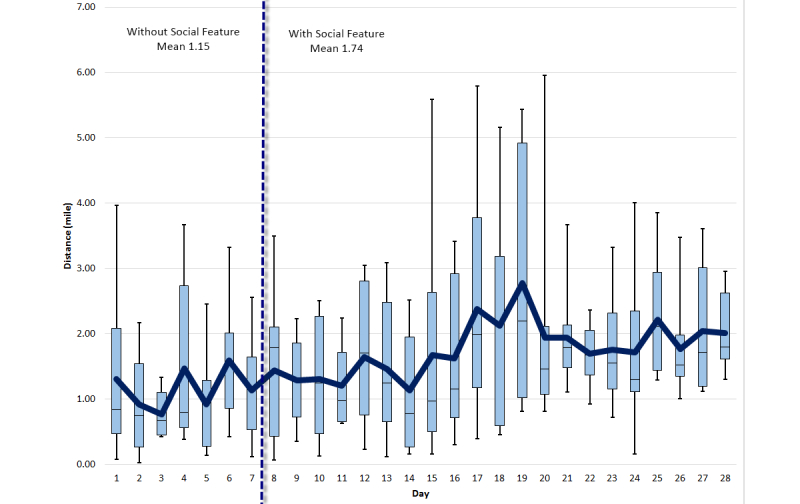
Distance comparison between baseline and social intervention.

**Figure 9:**
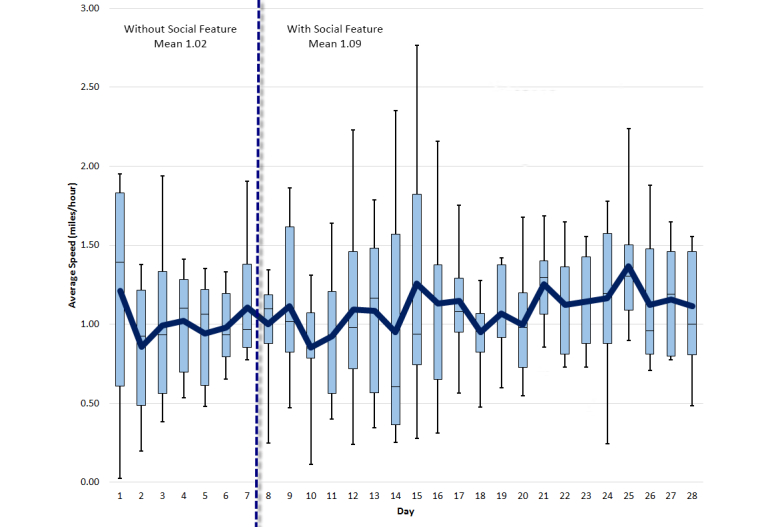
Average speed comparison between baseline and social intervention.

**Figure 10:**
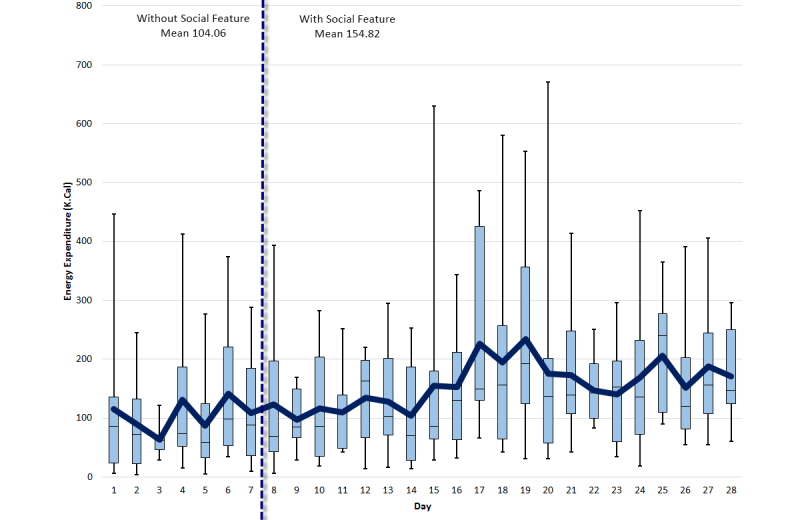
Energy expenditure comparison between baseline and social interaction.

**Figure 11:**
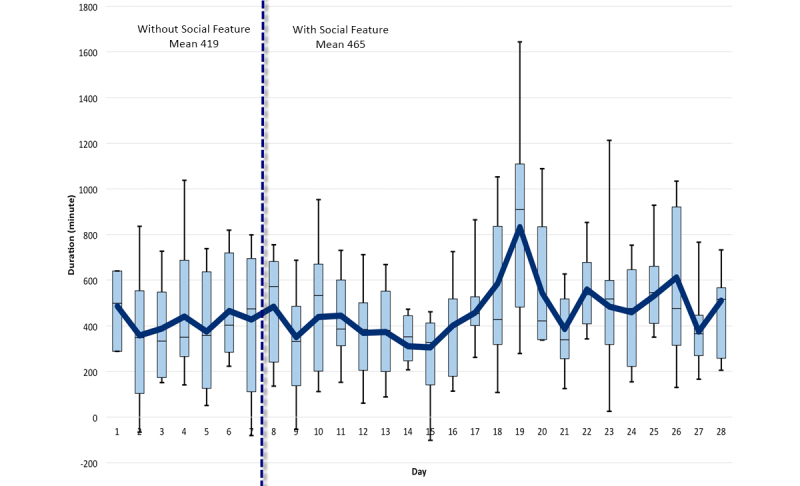
Duration of system usage comparison between baseline and social intervention.

An average difference of 2150 steps was recorded on average, per day, per participant for the first week, and all the following weeks. Figure 7 shows a detailed step-by-step comparison for all weeks. Figures 8-10 show other PA data comparisons; Figure 11 shows a system usage data comparison.

#### Social Interaction and Number of Steps

**Figure 12:**
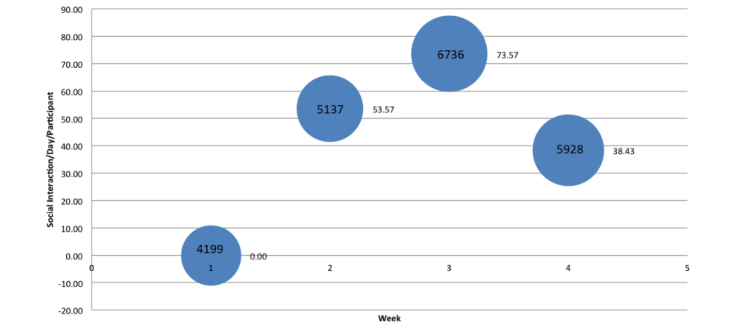
A plot of duration, social interaction number, and step number.

No trends were apparent in the relationship between the number of average steps/day/participant and social interaction. Figure 12 shows a bubble chart to plot the time, average steps/day/participant, and average social interaction/day/participant.

## Discussion

### An Important Goal

An important goal of this research was to develop an app that can monitor PA levels and effectively encourage users to engage in more PA. To that end, we need to ensure that the app complies with the established health behavior change theories and strategies, as well as delivers effective interventions. The PersonA Characteristics Model depicts the necessary characteristics needed for such a PA promotion app. Each individual and/or a combination of the characteristics on the model have been successfully implemented in many studies, and have given positive impact so that the model can be used as a blueprint and simple guideline by developers to build a system for PA promotion. In this study, the PersonA Characteristics Model was used as a foundation to develop the PersonA app. After the PersonA was developed, a typical usability study was conducted to find out whether it is usable and can be accepted by users. As a result, participants gave a high score for each factor of usability (ie, learnability, efficiency, error recovery, and subjective satisfaction), with an average of 4.52 out of a 5.00 maximum. Even with the small sample size of this pilot study, and no other apps to serve as comparisons for direct testing, the usability results suggest that the system is usable and that users were satisfied and enjoyed using it.

We also examined the feasibility of using PersonA for daily life PA promotion from a technological perspective. The specific purposes of the feasibility evaluation were to explore users’ experiences with the system, to determine the acceptability of the interventions and protocols, and to reveal other technology deployment issues to prepare for larger scaled studies and/or clinical trials. The quantitative analysis of this study demonstrated positive results. The dropout rate of this study was 7% (1 of 14), which is within the average dropout range of 4% to 16% reported by a meta-analysis of PA interventions [86], and is better than the 20% of another meta-analysis [87]. With regards to adherence, participants used the system for an average of 454 minutes (7.57 hours) per day per participant with SD 1.56 hours, and they accessed various system features an average of 28 times per day (SD 7.2). These numbers were high when compared with use numbers from a survey conducted by the Consumer Health Information Corporation. This survey found that smartphone apps have a high rate of dropout, with 26% being used only once, and 74% being discontinued by the 10th use [88]. The high usability scores, the high frequency of use, and the usefulness scores indicate that participants not only liked the design of the app, but also found it convenient, useful, and used it daily. The numbers also indicate that users would likely continue to use it long term, as it has been established that for a user to adopt and frequently use a smartphone app long term, the user must consider it both usable and useful [89,90]. This is consistent with the fact that lack of usability and usefulness are top reasons for users to discontinue smartphone app usage [88].

### Limitations and Strengths

The qualitative analysis identified a few utility limitations, as well as highlighted the acceptability of different parts of the intervention and its protocol. We identified that PersonA has some utility limitations, such as limited battery life, limited accuracies, simple measures, limited placement of the devices, and recording only user walking and running [84]. For example, to get an accurate number of steps, the smartphone was required to be placed on the hip (with a belt clip) or in the front pocket of the pants. We identified that this limited placement might impact the utility of the app, especially for female participants who tend to place their smartphone in their bags. Nevertheless, the qualitative analysis also highlighted the acceptability of PersonA to be used in daily life. The acceptability is indicated in the participants’ comments expressing that it helped them, to self-monitor their PA levels easily, to compare their performance with that of others, to facilitate a sharing experience, and to enable them to support each other. Thus, most participants also answered that they were willing to use PersonA if it became available in the future.

### Thematic Analysis

The thematic analysis of the qualitative data indicates that PersonA sometimes acted as a virtual coach, motivating a portion of the participants to be more physically active. Moreover, in looking at the participants’ perspectives, it appears that the combination of self-management practices and social support may act synergistically to keep some of them working toward their goals to have a more active lifestyle. New areas of inquiry were also identified during qualitative analysis, including: (1) the need to refine sources of motivation to use PA promotion, (2) to explore emergent health behaviors in response to smartphone-based health apps (such as users’ preference of the mobile over the Web version, if both are available), and (3) to explore users’ preferences as to data visualization type.

### PersonA and Social Interaction

The qualitative analysis also indicated that the social interaction on PersonA had various effects on the individual participants. These ranged from feeling pressured about PA, to feeling neutral, to feeling encouraged to do more PA. These different effects of social interaction on PA performance are consistent with the findings in other studies: (1) it increases PA performance [14,16,17,19,21]; and (2) it did not increase average PA performance, but did reduce participant attrition [83]. A possible explanation for this spectrum is that the effect of social interaction on PA performance is affected by personality type. Halko and Kientz [91], in a study, recognized such an association between the effect of persuasive technology, like PersonA, and personality type.

### Future Studies

In the future, PersonA’s validity evaluation should be the first priority because an inaccurate measurement in PA monitoring and promotion program has the potential to lead toward ineffective programs/support; frustration from the lack of results, and an inappropriately placed belief that increasing activity does not result in improved health outcomes. In addition, such an evaluation will give the system greater credibility, which will yield greater persuasive effects [92]. A potential method that can be applied for the evaluation is a comparison against other step monitoring devices, especially the widely known and more accurate pedometer. Until now, that is the most feasible method to validate a steps counter over a long duration in free-living conditions, as was done in three previous studies [93-95].

To fully elucidate the potential benefit of PersonA in increasing PA levels, long term and large sample size randomized control trials in the outpatient setting are required. Such trials should include heterogeneous participants in terms of age, gender, socioeconomic status, personality type, and experience with SNS and smartphones. A similar trial with a more appropriate research design (eg, baseline-intervention or randomized controlled trial) should also be conducted to explore the association between online social interaction and PA performance. This future trial may lead to the development of effective social interaction techniques, and the exploration of effective methods of ecological intervention using a SNS. Last, the online social interactions in this PersonA study included two or more types of social interactions (viewing others’ data, comparing data, sending messages, receiving messages, etc), so that the independent contribution of any one of these components is difficult to establish. Hence, a more detailed and structured study to examine the effects each type of social interaction has on PA performance is also warranted.

To examine usability, the sample size of this study appears appropriate according to the Problem Discovery Rate Model [76-78]. Nonetheless, the number for this study seems to be a bit low when taking into account the fact that it was a homogeneous sample population. Our participants tended to be adult, female, college educated, and already experienced with the technologies used in PersonA (smartphones and online social interaction). Additional research would be needed to determine whether the findings extend to a demographically more heterogeneous sample, and to those who have no prior experience with smartphones and social interaction technologies. A similar theme arises when evaluating the feasibility of using PersonA in PA promotion. The results from the quantitative and qualitative analyses demonstrate that deploying PersonA with self-management and social network features to promote PA in daily life is feasible. Nonetheless, these results should be interpreted with caution because of the study limitations: (1) the small size and homogeneous characteristics of the sample, (2) the short term duration of study, (3) no other apps as a comparison, and (4) unstandardized and invalidated outcome measures. Thus, the findings are not conclusive and will require validation from a larger trial study with a more representative population.

## Abbreviations

4G: 4th generation
API: application-programming interface
app: applications
CBCI: common bond and common identity theory
ELM: Elaboration Likelihood Model
FBM: Fogg Behavioral Model
HBM: Health Belief Model
IT: information technology
JSON: JavaScript Object Notation
OS: operating system
PA: physical activity
PersonA: Persuasive Social Network for Physical Activity
POI: point of input
PSD: Persuasive System Design
PSSUQ: International Business Machine Post Study System Usability Questionnaire
SCT: social cognitive theory
SNS: social networking system
TAM: Technology Acceptance Model
TPB: theory of planned behavior
TRA: theory of reasoned action
UGT: uses and gratifications theory
UTAUT: Unified Theory of Acceptance and Use of Technology
